# FRET Ratiometric Nanoprobes for Nanoparticle Monitoring

**DOI:** 10.3390/bios11120505

**Published:** 2021-12-09

**Authors:** Guangze Yang, Yun Liu, Jisi Teng, Chun-Xia Zhao

**Affiliations:** 1 Australian Institute for Bioengineering and Nanotechnology, The University of Queensland, Brisbane, QLD 4072, Australia; g.yang@uq.edu.au (G.Y.); yun.liu2@uq.net.au (Y.L.); tengjisi@uq.net.au (J.T.); 2 ARC Centre of Excellence for Enabling Eco-Efficient Beneficiation of Minerals, The University of Queensland, Brisbane, QLD 4072, Australia; 3 School of Chemical Engineering and Advanced Materials, Faculty of Engineering, Computer and Mathematical Sciences, The University of Adelaide, Adelaide, SA 5005, Australia

**Keywords:** microfluidics, nanoparticles, nanomedicine, drug delivery, lipid, polymer

## Abstract

Fluorescence labelling is often used for tracking nanoparticles, providing a convenient assay for monitoring nanoparticle drug delivery. However, it is difficult to be quantitative, as many factors affect the fluorescence intensity. Förster resonance energy transfer (FRET), taking advantage of the energy transfer from a donor fluorophore to an acceptor fluorophore, provides a distance ruler to probe NP drug delivery. This article provides a review of different FRET approaches for the ratiometric monitoring of the self-assembly and formation of nanoparticles, their in vivo fate, integrity and drug release. We anticipate that the fundamental understanding gained from these ratiometric studies will offer new insights into the design of new nanoparticles with improved and better-controlled properties.

## 1. Introduction

Fluorescence imaging has been widely used in monitoring nanoparticle drug delivery. Nanoparticles (NPs) are either loaded with fluorescent dyes inside or labelled with fluorescent dyes on the particle surface [1,2]. Then, NP uptake or biodistribution can be quantified by monitoring the fluorescence signal in vitro or in vivo. The underlying assumption is that the fluorescence signal of NPs has a positive correlation with their accumulation in cells, tissues or organs. However, the absolute intensity of a single fluorescence dye is often inaccurate and non-comparable because many factors could affect its intensity, including background signal, instrumental set-up, and materials screening and quenching. Furthermore, the fate of NPs in vitro or in vivo cannot be accurately monitored by using fluorescence-dye-labelled NPs, as the dye might be already separated from the NP itself due to release or degradation [3,4]. Therefore, single fluorescence probes cannot be used for the accurate monitoring of NP drug delivery.

Förster resonance energy transfer (FRET) describes an energy transfer between two fluorophores, a donor and an acceptor. In an excited state, the donor fluorophore transfers energy to the acceptor fluorophore. The efficiency of this energy transfer is inversely correlated to the distance between these two fluorophores. Consequently, energy transfer can only occur in the 1–10 nm range [5]. Therefore, optical fluorescence imaging by taking advantage of the FRET phenomenon provides a distance ruler to characterize the properties of nanoparticles and their interactions with biological systems [6]. FRET offers a powerful tool to monitor NP drug delivery including NP integrity, drug release, NP degradation or swelling, which are critical to fundamentally understand the in vitro and in vivo fate of NPs, thus providing critical information for their drug delivery applications [7,8]. Many fluorescence dyes are suitable for in vitro studies; however, it is more challenging for in vivo imaging because of the intrinsic limitations such as poor tissue penetration, and strong background fluorescence signals. In contrast, near infrared (NIR) fluorophores provide a sensitive probe for in vivo imaging [9].

**Scheme 1:**
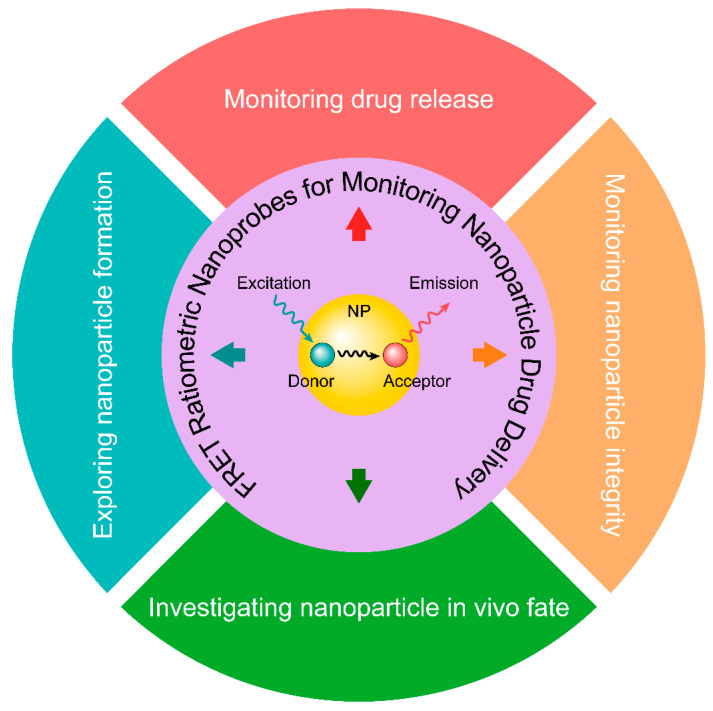
Overview of the structure of this review. This review summarizes the different designs of FRET ratiometric nanoprobes for monitoring NP drug delivery (inner ring). The FRET basics (inner core) are introduced, then various FRET applications for drug delivery, including exploring nanoparticle formation, investigating nanoparticle in vivo fate, monitoring nanoparticle integrity, and monitoring drug release are reviewed (outer ring; blue, green, orange, and red colours, respectively).

This article reviews different designs of FRET ratiometric nanoprobes for monitoring NP drug delivery. We start with a brief introduction about different FRET pairs and their properties including FRET basics, FRET efficiency, FRET ratio, and FRET pairs. Then, we discuss the applications of FRET for monitoring NP formation or NP self-assembly, for investigating NP in vitro or in vivo fate, and for exploring NP integrity and NP drug delivery. We provide a critical review of deploying FRET ratiometric nanoprobes for fundamentally understanding many important aspects of using NPs for drug delivery (Scheme 1). The design of different FRET nanoprobes offers a powerful tool to gain insight into NP formation, and NPs’ interaction with biological systems in vitro and in vivo.

## 2. Different FRET Pairs and Their Properties

### 2.1. FRET Basics

FRET requires an integral overlap between the emission spectrum of the donor fluorophore and the excitation spectrum of the acceptor fluorophore. For FRET to occur, emission from the donor must be able to excite the acceptor, i.e., the donor fluorescence (F_D_) overlaps the acceptor excitation spectrum (ε_A_). This overlap is quantified by the spectral overlap integral J (in units M^−1^ cm^−1^ nm^4^), as shown in Equation (1) [6]:

(1) $$J\left(\lambda \right)=\frac{\int_{0}^{\infty }F_{D}\left(\lambda \right)\varepsilon_{A}\left(\lambda \right)\lambda^{4}d\lambda }{\int_{0}^{\infty }F_{D}\left(\lambda \right)d\lambda }$$

A minimum of 30% overlap is essential for an efficient FRET. Additionally, a close distance of 1–10 nm between the donor and acceptor fluorophores is required for the formation of FRET. Theoretically, FRET efficiency (E) describes the fraction of photons transferred from the donor to the acceptor, so it is strongly dependent on the distance between the donor and acceptor (R), as shown in Equation (2) [10],

(2) $$E=\frac{R_{0}^{6}}{R_{0}^{6}+r^{6}}$$

where r is the actual distance between the two fluorophores, and R_0_ stands for the Förster radius. R_0_ corresponds to the distance at which E equals 50%, and it correlates with the spectral overlap between the emission of the donor fluorophore and absorbance of the acceptor fluorophore. For most FRET pairs 1 nm < R_0_ < 10 nm, R_0_ depends on J and the donor quantum yield ($\phi_{D}$) [6]:

(3) $$R_{0}=0.0211{\left(J\phi_{D}k^{2}n^{-4}\right)}^{\frac{1}{6}}$$

where k^2^ in Equation (3) is the orientation factor, which depends on the relative orientation of the donor and acceptor transition dipole moments. The parameter n is the refractive index between the donor–acceptor pair. In practice, E can be maximized by the donor–acceptor selection of the FRET pair dyes based on their optical properties [6].

FRET depends strongly on the distance between the donor dye (D) and acceptor dye (A). Therefore, FRET pairs can be incorporated into NPs at different positions to monitor their formation, in vivo fate and integrity, and NP degradation and drug release. When the FRET pair are in close proximity within NPs, energy transfer is successful from D to A, resulting in D quenching and A emission. Upon NP degradation and dye release, the FRET signal decreases along with the increase in the D signal due to the increased distance between D and A. FRET ratio is often used for quantitative analysis. It is defined as the ratio of the fluorescence intensity of an acceptor (F_A_) to the total fluorescence intensity of the donor and the acceptor (F_A_ + F_D_), each recorded in its emission wavelength using an excitation wavelength of the donor as defined below [11,12]:

(4) $$\mathrm{FRET}\;\mathrm{ratio}=\frac{F_{A}}{F_{A}+F_{D}}$$

For in vivo experiments, the intensity of donor and acceptor signals should be corrected from the autofluorescence background measured before NP uptake in cells or injection in animal. The FRET ratio provides a semi-quantitative way to correlate it with particle integrity.

### 2.2. Different FRET Pairs

**Figure 1:**
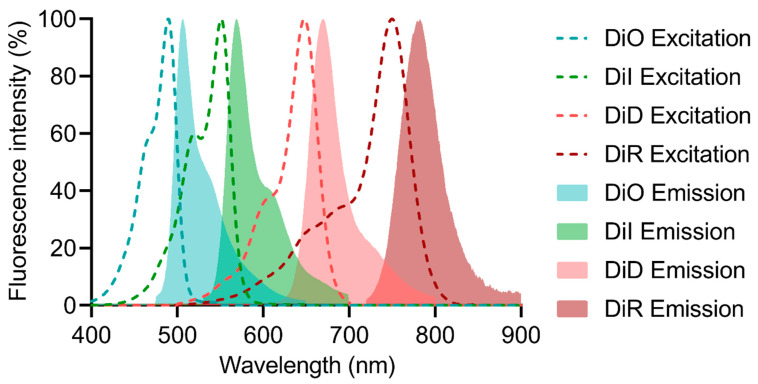
Fluorescence spectra of lipophilic tracer dyes.

Lipophilic tracers are widely used as FRET pairs including 3,3′-dioctadecyloxacarbocyanin perchlorate (DiO), 1,1′-dioctadecyl-3,3,3′,3′- tetramethylindocarbocyanine perchlorate (DiI), 1,1′-dioctadecyl-3,3,3′3′-tetramethylindocarbocyanine perchlorate (DiD), and 1,1′-dioctadecyl-3,3,3′,3′-tetramethylindotricarbocyanine iodide (DiR) (Figure 1). DiO and DiI are often used as a FRET pair for in vitro cell experiments due to their short excitation wavelengths and weak tissue penetration. DiD and DiR are widely used as a near infrared (NIR) FRET pair for in vivo animal or zebrafish studies because they have minimal interference of absorbance and fluorescence signals from tissue samples and enhanced tissue penetration.

**Figure 2:**
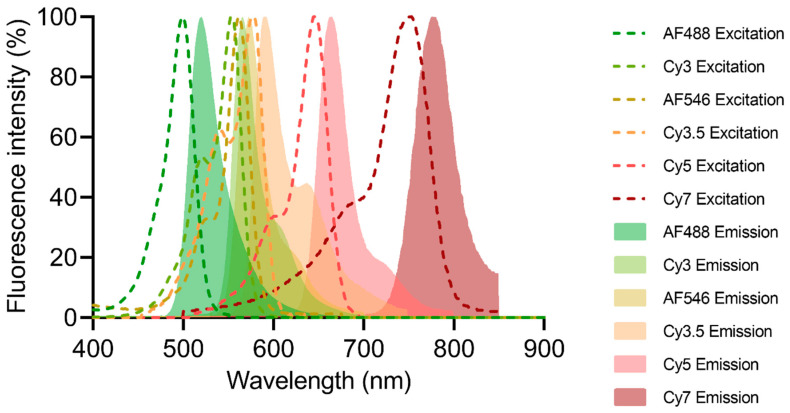
Fluorescence spectra of cyanine and Alexa Fluor dyes.

Fluorescein isothiocyanate (FITC) and Rhodamine B is a classical FRET pair with the donor’s (FITC) excitation/emission of 495/517 nm and the acceptor’s excitation/emission of 543/565 nm. Cyanine (Cy) dyes are another type of fluorophore (e.g., Cy3, Cy5, Cy5.5, Cy7, Cy7.5) that have been widely selected as FRET pairs for in vitro and in vivo studies (Figure 2). They offer advantages such as high fluorescence stability, and good biocompatibility compared with the FITC/Rhodamine B pair. Alexa Fluor dyes are an alternative to the bulky Cy dyes, such as Alexa Fluor 488 and Alexa Fluor 546, and they have high fluorescence quantum yield, high photostability, and great sensitivity due to their limited self-quenching even at high molar ratios when they are attached to molecules or particles, thus enabling more sensitive detection.

For FRET studies, when an excited donor (e.g., nanoparticle, ion, a molecule, or molecular complex) transfers the excitation energy non-radiatively to an acceptor, both donor and acceptor are considered as point dipoles [13]. The electronic dipole–dipole coupling of donor and acceptor molecules can improve the FRET efficiency. Consequently, it has gained potential applications in many fields of science and technology lately to use metal nanoparticles in combination with fluorescent probes. Several studies have reported that metal nanoparticles of different shapes and sizes can significantly affect the fluorescence of the fluorophore within an ideal distance [14,15]. For instance, one study reported the application of FRET between fluorescent probe (biologically active coumarin derivative 2-acetyl-3H-benzo[f]chromen-3-one) and microwave-assisted silver nanoparticles in propanol solvent at room temperature [15]. Additionally, gold nanoparticles can also be exploited as a good energy transfer acceptor for manufacturing biosensors due to their distinctive electrodynamic properties. Their characteristic plasmon resonance can spectrally overlap with the emission of a variety of donors [13].

Self-quenching is one of the critical issues in FRET imaging, which can be explained by an intramolecular ground-state dimer complex between the FRET pair in an aqueous solution. The molecular structure, solvent, the presence or absence of electrolytes, and the temperature of FRET pair dyes can affect their quenching degree [16]. It is vital to reduce the self-quenching of the donor to increase the energy transferred from the donor to the acceptor. It was reported that a lower number of dye molecules was more beneficial for better light-harvesting in surface-functionalized nanoparticles [17]. In contrast, J-aggregate is one type of self-quenching, which is formed by a highly ordered assembly of organic dyes [18]. The strong shift of their absorption and emission spectra to longer wavelengths of J-aggregates allows the real-time monitoring of the quenching degree of organic dyes. Nanoparticles with high loading of FRET pairs exhibited not only the FRET phenomenon but also a J-aggregate red shift. Therefore, the J-aggregate-based FRET approach is a useful tool for investigating the structure and release of nanoparticles with high drug or dye loading [19].

In addition to popular FRET pairs as discussed above, many other fluorescent molecules or particles such as fluorescent proteins (FPs, e.g., GFP), quantum dots (QDs) and upconversion NPs have been developed as FRET pairs (Table 1). For example, an efficient FRET occurs between near-infrared (NIR)-responsive lanthanide-doped upconversion NPs (UCNPs) and Fenton reagent ferrocenyl compounds (Fc) [20]. The applications of semiconductor nanocrystals as FRET donors have been increasing over the past years, such as the QD-Cy5 and QD-Cy7 systems [21]. Drug carriers with aggregation-induced emission (AIE) dyes can be used as AIEgen-drug FRET pairs to monitor drug delivery and drug release using both AIE and FRET effects. For instance, one study used the hyperbranched polyamide amine with intrinsic AIE effects as the core, and achieved the dynamic tracking of drug carriers and the dynamic drug release of DOX [22]. There is no one-size-fits-all probe for all applications, and different probes have their distinct advantages and disadvantages that need to be carefully considered and selected for different studies [23].

## 3. FRET for Exploring Nanoparticle Formation

Many NPs are generated through self-assembly, such as lipid-based NPs, polymeric NPs or micelles [51,52]. An improved understanding of the NP self-assembly process facilitates the future design and preparation of NPs with better-controlled properties, and also improves the understanding of the structure of the generated NPs.

**Figure 3:**
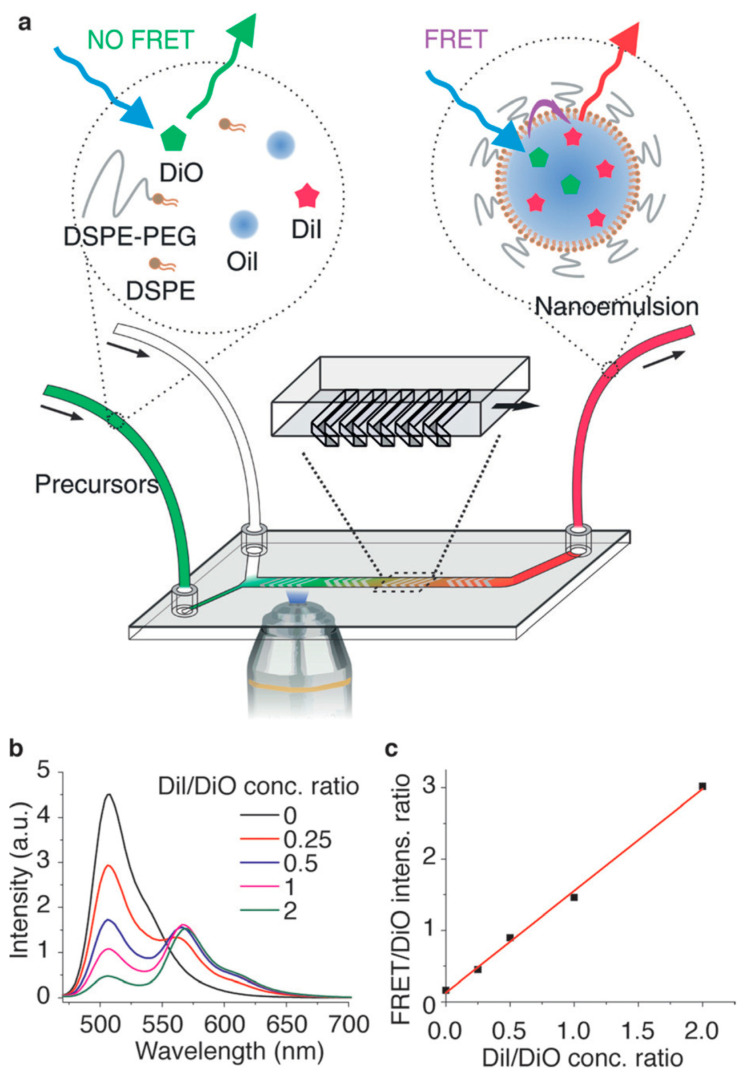
Real-time monitoring of nanoemulsion formation using FRET in a microfluidic device. (**a**) Schematic of the experimental setup and FRET mechanism. (**b**) Emission intensity of nanoemulsions containing different DiI/DiO concentration ratios. (**c**) Correlation between the FRET/DiO intensity ratio and the DiI/DiO concentration ratio. Reproduced with permission from ref. [25]. Copyright 2017, Wiley-VCH Verlag GmbH & Co. KGaA, Weinheim, Germany.

FRET in combination with microfluidics enables the visualization of the NP formation process (Figure 3) in real time, including nanoemulsions, polymeric micelles and high-density lipoprotein NPs. To monitor nanoemulsion formation, an ethanol solution containing a lipid mixture of 1,2-distearoyl-sn-glycero-3-phosphocholine (DSPC), 1,2-distearoyl-snglycero-3-phosphoethanolamine-N-[methoxy(polyethyleneglycol)-2000] (DSPE-PEG2000), and cholesterol, and medium-chain triglycerides along with a FRET pair DiO and DiI was mixed with an aqueous solution in a Herringbone mixer microfluidic device at a volume ratio of 1:4. Upon fast mixing in the device, lipids self-assembled and oil aggregated, leading to the formation of nanoemulsions. During this self-assembly process, the FRET pair (DiO and DiI) was spontaneously incorporated into the oil core of the nanoemulsion due to their intrinsic hydrophobicity, triggering the increase in the FRET signal and the decrease in the donor DiO peak (Figure 3). The FRET/DiO intensity ratio was shown to have a linear correlation with the DiI/DiO concentration ratio, and thus can be used to monitor the nanoemulsion formation [25].

**Figure 4:**
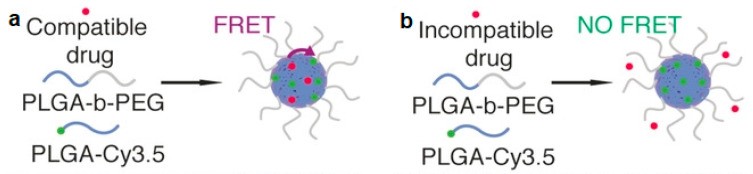
Real-time monitoring of drug loading and FRET mechanism of PLGA-b-PEG polymer NPs using (**a**) a hydrophobic Cy5-OLA model drug and (**b**) a hydrophilic sulfo-Cy5 model drug. Reproduced with permission from ref. [25]. Copyright 2017, Wiley-VCH Verlag GmbH & Co. KGaA, Weinheim, Germany.

The FRET imaging method was also applied to visualize the NP assembly and drug-loading process using poly(lactic-co-glycolic acid)-block-poly(ethylene glycol) (PLGA-b-PEG) block copolymers. The donor dye Cy3.5 was conjugated to the distal end of the PLGA chain, and Cy5 dye was used as a model drug. FRET occurs only when the Cy5 model drug is incorporated into the Cy3.5-labelled PLGE-PEG NPs, resulting in the increase in the FRET/Cy3.5 intensity ratio (Figure 4). PLGA-b-PEG block copolymer, PLGA-Cy3.5, and Cy5 were firstly dissolved in acetonitrile which was then mixed with PBS in a microfluidic chip. At the end of the microfluidic channel, a high FRET/Cy3.5 intensity ratio was observed, indicating the successful encapsulation of Cy5 into the NPs. In contrast, sulfo-Cy5 (logD = −5.5 at pH 7.4) failed to be incorporated in the NPs due to its high water solubility, as demonstrated by the unchanging FRET/Cy3.5 ratio [25]. Furthermore, FRET was used to monitor the formation of QD-loaded high-density lipoprotein (QD-HDL) NPs using 600 nm-emitting phospholipid-coated QD (PL-QDs) and Cy5-labelled apolipoprotein A1 (APOA1-Cy5). The formation of QD-HDL NPs was not instant (seconds to minutes) probably due to the slow diffusion of PL-QDs and APOA1, so a microfluidic device with an extended loop is required for imaging [25].

FRET is adopted to explore the assembly and disassembly of thermosensitive “micellar hydrogel” using poly(ε-caprolactone-co-1,4,8-trioxa[4.6]spiro-9-undecanone)-b-poly(ethylene glycol)- b-poly(ε-caprolactone- co-1,4,8-trioxa[4.6]spiro-9-undecanone) (PECT) triblock copolymer. A FRET pair, fluorescein isothiocyanate (FITC, donor) and rhodamine B (RB, acceptor), was conjugated separately to PECT polymers (PECT–FITC and PECT–RB, respectively). When the PECT–FITC and PECT–RB are co-assembled to form micelles, the FRET pair is able to be locked in the micelles within a distance range of 10 nm, showing a strong FRET signal. Any micelle disassembly could lead to changes in FRET ratio [39]. The FRET ratio of the micelles co-assembled by PECT–FITC/PECT–RB/PECT polymers remained the same during 21 days of incubation, confirming that PECT hydrogel formation was driven by the temperature-responsive aggregation of micelles, and the PECT polymers were steadily incorporated in the micelles without any polymer interchange, re-assembly or micelle disintegration [39].

FRET has also been explored to monitor the formation of core–shell NPs. Khalin et al. created polymer poly(methyl methacrylate)-sulfonate (PMMA-SO_3_H) NPs with stealth properties using the amphiphilic block copolymers pluronic F-17 and F-68 to prevent NPs from nonspecific interactions with serum proteins. To confirm the formation of a stable shell on the particle shell, FRET signal was monitored between the donor dye (rhodamine B) in the PMMA-SO_3_H NPs and the acceptor dye (Cy-5) conjugated with pluronic PF-127 [40].

FRET is a useful tool to investigate drug co-encapsulation. The co-loading of curcumin with a natural potent photosensitizer and a promising photodynamic therapy candidate hypericin (HYP) into low-density lipoproteins (LDL) were examined using FRET. LDL NPs with CUR loaded at two different concentration ratios (CUR: LDL 3:1 and 30:1) were prepared. HYP was incorporated into these two formulations. FRET was monitored between CUR and HYP by exciting the donor CUR. The FRET signal confirmed the monomeric form of HYP molecules inside LDL NPs. With the increase in HYP concentration, the fluorescence intensity of CUR decreased, while the fluorescence intensity of HYP increased [41].

FRET is a powerful tool to observe the formation of NPs, which is difficult to be achieved via other approaches. By either loading or conjugating FRET fluorophores inside NPs or to self-assembling molecules, direct visualisation of NP formation can be realised through microfluidic devices. This will provide valuable information such as NP formation time, encapsulation efficiency, coating efficiency, etc.

## 4. FRET for Investigating Nanoparticle In Vivo Fate

NPs are normally labelled with fluorescence dyes to track their biodistribution and in vivo fate. However, the dye molecules either encapsulated inside NPs or conjugated on NP surface might experience different changes over time after administration, for example, dye release, and separation of the dye from NPs, which lead to misleading results. FRET can be used as a more accurate method to monitor the biodistribution and in vivo fate of NPs. Callmann et al. synthesized enzyme-responsive micellar NPs modified with a peptide (GPLGLAGGERDG) targeting the matrix metalloproteinases (MMPs), which are overexpressed in many cancer types, and a hydrophobic paclitaxel core using paclitaxel-conjugated di-block copolymers. A FRET pair, fluorescein and rhodamine, was formulated into a single NP via grafting to the second block of the polymer, respectively, to enable tracking the in vivo fate of these NPs. With both responsive (NP-L) and non-responsive (NP-D) NPs, FRET was observed up to 5 d following IV injection of NP-L, suggesting the accumulation and retention of these materials over a long time. In contrast, FRET was only observed for the first 5 h following IV injection of NP-D, indicating a rapid clearance. Additionally, tumour growth was suppressed by NP-L up to 12 d post injection. The targeting capabilities of NP-L were analysed by monitoring the FRET signal at the tumour site. A FRET signal appeared at the tumour within 3 h post injection, and lasted for up to 3 d [53]. Liu et al. synthesized liposomes labelled with a FRET pair (DiR and DiD) for in vivo pharmacokinetics studies, revealing that they were able to enhance oral drug absorption, by either extending their contact time with the gastrointestinal tract or improving their penetration through the mucus barrier, rather than being absorbed intact into blood circulation [38]. Carye et al. used a Cy5.5 and Cy7.5 FRET pair to monitor the in vivo fate of NPs made of the squalene–gemcitabine prodrug [30]. Cy5.5 and Cy7.5 were conjugated to squalene, and the squalene drug and squalene dye self-assembled to form NPs. After administration, the NPs accumulated at mouse liver and showed strong FRET fluorescence. Starting from 2 h, the FRET signal gradually decreased at the liver, and the fluorescence of both FRET and the donor finally disappeared at 48 h, demonstrating the clearance of the NPs.

Mammalian mice models are commonly used animal models to study NP transport, but they are expensive and time consuming. Zebrafish (Danio rerio) has emerged as a cost-effective and valuable “intermediate” vertebrate model for quick evaluations of NP drug delivery and in vivo fate of NPs due to the similarity of structure and function of the biological barriers between zebrafish and mammals at certain levels. For example, zebrafish blood–brain barrier (BBB) function is developed at 3 days post fertilization(dpf), and it acts as a barrier restricting the permeability of many compounds, which is similar to the BBB barrier in humans. The nanocrystals coumarin 6/DiI-NCs were produced by mixing C6 and DiI at a 1:1 molar ratio. When diluting the C6/DiI-NCs in water, strong FRET occurred with a FRET ratio of 0.94. However, FRET disappeared when dispersing them in methanol, due to the dissolution of NCs, and thus the separation of the FRET pair. Larval zebrafish (7 dpf) were used to probe the in vivo fate of NCs. No obvious fluorescence was observed in the first 5 min. However, the FRET signal (red) increased over time, indicating the transport of intact NCs in zebrafish. At the same time, green fluorescence appeared and grew with the incubation time, demonstrating that some NCs gradually disassociated [42].

The energy transfer process leads to the quenching of the donor fluorescence, which may induce a shortening of its lifetime. Therefore, the lifetime-dependent FRET can also be used to study nanoparticle fate. One of the advantages of using the lifetime-dependent FRET approach is that the lifetime FRET imaging only needs a single image from the FRET sample at the donor excitation and emission wavelengths to extract the energy transfer parameters, with a donor-only sample as the negative control [54]. The other advantage of the lifetime-dependent FRET approach is that it allows differentiating the free and associated donor molecules [55]. The lifetime-dependent FRET approach has been exploited to investigate the protein–protein interaction, and FRET can occur between proteins labelled with proper donor and acceptor fluorophores. As a result of successful FRET, both the intensity and the lifetime of the donor fluorescence decline, while the intensity of the acceptor emission rises. Therefore, the FRET efficiency can be measured by either the intensity changes in the donor and acceptor emission or the changes in the lifetime of the donor molecule. One study reported the application of the lifetime FRET approach to imaging protein–protein interactions in live cells between the epidermal growth factor receptor (EGFR) and the adapter protein Grb2 [56]. Another study developed biosensors with red-shifted fluorescent proteins, which usually have a longer fluorescence lifetime than that of GFP, which should improve the dynamic range of the biosensor for time-resolved (lifetime) fluorescence measurements [57].

Single-dye-labelled NPs have been extensively investigated for understanding the in vivo fate of NPs. However, due to the complexity of the in vivo environment, they may provide inadequate or inaccurate information. FRET-labelled NPs have emerged as a useful tool to further understand the bio–nano interactions in vivo in recent years. The dynamic changes in both donor and acceptor signals can significantly improve the accuracy and provide more information for the in vivo fate of NPs, such as dye release, NP degradation, and NP clearance.

## 5. FRET for Monitoring Nanoparticle Integrity

NP needs to be stable and remain intact during circulation, and release the loaded drug at the disease site. FRET offers a facile and powerful tool to monitor NP integrity in vitro and in vivo. Encapsulation of a FRET pair in NPs should give a high FRET signal, but disassembly or disintegration of NPs will result in the decrease or loss of the FRET signal depending on the extent of disintegration. As FRET occurs between two fluorophores within close proximity, the dual emission signals allow quantitative ratiometric measurement in two optical windows. Furthermore, a quantitative correlation between the FRET ratio and NP integrity could be established, allowing the monitoring of NP integrity in real time based on the change in FRET ratios. FRET imaging has been successfully applied to investigate the biodistribution and integrity of various NPs, including polymer NPs, micelles, lipid NPs, and nanoemulsion droplets.

**Figure 5:**
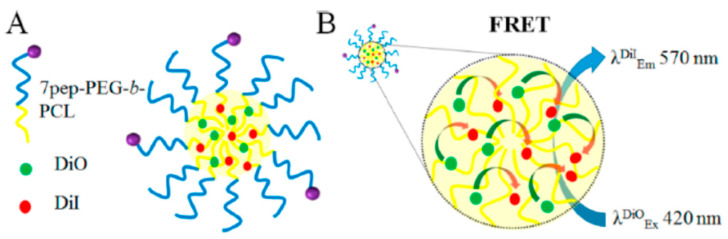
Schematics of the FRET micelle (**A**); concept of the FRET micelles (**B**), FRET mechanism of the DiO- and DiI-loaded micelles. Reproduced with permission from ref. [12]. Copyright 2017, American Chemical Society.

Polymer micelles are one of the most investigated nanocarriers for drug delivery, but they tend to disassemble upon quick dilution after systematic administration in vivo. Fundamental understanding of their interaction with biological systems and their in vivo fate is important. Li et al. fabricated 7peptide (7pep)-decorated poly-(ethylene glycol)-block-poly(ε-caprolactone) micelles with different ligand densities for targeted delivery. The 7pep (HAIYPRH) peptide exhibits high affinity to TfR, a trans-membrane receptor, that is expressed on intestine, brain, eye, lung, etc. A FRET fluorophore pair (DiO and DiI) was co-loaded into the 7pep−M−C6 PCL micelles at a 1:1 molar ratio (Figure 5). When the micelles were incubated in the cell culture medium E3, they were stable, as indicated by the FRET ratio of 47.18%. However, when they were dispersed in acetone, the FRET ratio decreased to 10.80%, indicating the dissolution of the micelles in the hydrophobic solvent. For in vivo experiments in adult zebrafish, the micelles remained intact during the first 2 h, but started to disassemble during 2–4 h, as demonstrated by the signal decrease in red fluorescence from DiI [12].

Sun et al. further investigated the key reasons causing the disassociation of polymer micelles. They synthesized two polymer micelles of polyethylene glycol-block-poly(ε-caprolactone) (PEG-PCL) and PEG-block-poly(D,L-lactide) (PEG-PDLLA). A FRET pair, Cy5 and Cy5.5, was conjugated to the hydrophobic chain ends of the polymers to monitor their stability. PEG-PCL micelles dissociated to some extent in serum or blood, but most of them disassembled when shear was applied by a microfluidic channel. The FRET imaging experiments further demonstrated that both the PEG-PCL and PEG-PDLLA quickly disassociated into unimers, and then were sequestered in the Kupffer cells residing in the liver, mainly associated with albumin in the blood and liver. About 80% of the micelle disassociation was attributable to the shear and blood proteins, especially albumin, upon i.v. injection, while intact micelles were able to accumulate at the tumour site, and were then taken up and disassociated in cells [26].

**Figure 6:**
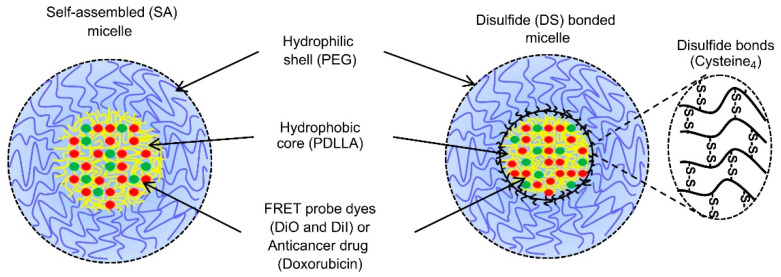
Schematic illustrations of self-assembled mPEG-PDLLA micelles and disulphide-bonded mPEG-(Cys)4-PDLLA micelles with FRET dyes (DiO and DiI). Reproduced with permission from ref. [11]. Copyright 2012, Elsevier Ltd., Amsterdam, Netherlands.

To control micelle stability, a disulphide bond could be incorporated into micelles to be responsive to glutathione (GSH), as the GSH concentration in some cancers is about seven times higher than that in normal cells, and its concentration in cytoplasm is also significantly higher (1–10 mM) than in extracellular fluid (1–10 µM). FRET imaging was used to study the stability of disulphide-bonded methoxypoly(ethylene glycol)-(cysteine)4-poly(D,L-lactic acid) (mPEG-(Cys)4-PDLLA) micelles. DiO and DiI were coloaded in the m-PEG-PDLLA micelles without disulphide bonds and mPEG-(Cys)4-PDLLA micelles with disulphide bonds (Figure 6). The FRET ratio of mPEG-PDLLA micelles decreased from 0.92 in water to 0.26 in DMSO. In contrast, the disulphide (DS)-bonded mPEG-(Cys)4-PDLLA micelles only had a moderate FRET ratio decrease, from 0.92 to 0.72, after adding DMSO. When incubated with 80% FBS solution at 37 °C, the FRET ratio of m-PEG-PDLLA micelles dropped from 0.95 to 0.63 due to the disassociation induced by serum proteins, such as globulins. In contrast, the FRET ratio of the mPEG-(Cys)4-PDLLA micelles in serum only decreased from 0.91 to 0.83, but quickly diminished to 0.55 upon incubation with GSH due to the cleavage of the disulphide bonds. The mPEG-PDLLA micelles disassembled rapidly in the bloodstream, as indicated by the quick decrease in FRET ratio from 0.58 at 15 min post injection to 0.42 at 6 h and a nearly complete loss of FRET at 12 h [11].

Different FRET strategies can be designed to investigate either micelle stability, biodistribution, or the disassociation of drugs from micelles. For example, empty FRET micelles assembled using poly(g-propargyl-L-glutamate) PEG-b-PPLG-Cy5.5 and PEG-b-PPLG-Cy7 were employed to probe their persistence, biodistribution, and stability. To examine the dissociation of drugs from micelles, PEG-b-PPLG-Cy7 formulations were prepared to encapsulate Cy5.5 as a model drug. The micelle carrier remained stable, with the FRET ratio remaining between 50 and 85% for up to 72 h with an initial FRET ratio of 94%. The micelles were able to circulate long [27]. Additionally, the micelles were demonstrated to be capable of retaining a significant portion of the encapsulated small molecular drug, as indicated by an oscillating FRET ratio of the Cy5.5-loaded PEG-b-PPLG-Cy7 between 28 and 40% [27].

**Figure 7:**
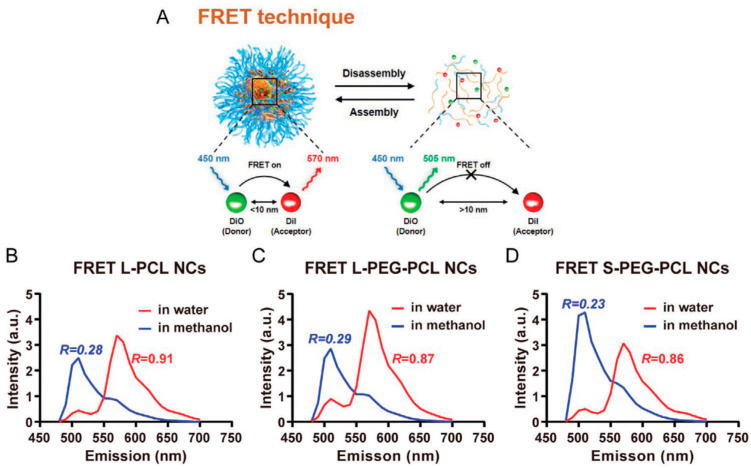
FRET for studying in vitro and ex vivo stability of polymeric NCs. (**A**) Schematic of the FRET principle (“FRET on”) and disappearance (“FRET off”). (**B**–**D**) Fluorescence spectra and FRET ratio of large PCL NCs (**B**), large PEG-PCL NCs (**C**), and small PEG-PCL NCs (**D**) in water (red) and methanol (blue). Reproduced with permission from ref. [58]. Copyright 2021, Wiley-VCH GmbH.

As an emerging model, transparent zebrafish offers a new in vivo system for real-time whole-body imaging. This model has many advantages, such as physiological homology with humans, easy genome editing, transparency, and high-spatiotemporal-resolution in vivo imaging. Therefore, the zebrafish has been employed for studying nano–bio interactions. Tao et al. attempted to correlate the NP elimination in zebrafish with rodents. They synthesized poly(caprolactone) nanocarriers (PCL NCs) with or without PEGylation using FRET [58]. A FRET pair, DiO and DiI, was co-encapsulated into the PCL NCs to study the NC stability (Figure 7). After intravenous injection of NCs in 7 dpf zebrafish larvae and mice, PEGylated PCL NCs showed higher integrity in both models, while PEG-PCL NCs with bigger particle sizes exhibited significantly higher macrophage uptake in zebrafish and in mice spleen, thus leading to poor circulation. Through dilution experiments in PBS, the FRET ratio of bigger PCL NCs (213.4 nm) and bigger PEG-PCL NCs (218.2 nm) decreased from 0.84 and 0.88 to 0.75 and 0.70, respectively, indicating that the dilution did not much affect the stability of these NCs. In contrast, rat serum destabilized the NCs dramatically, as indicated by the rapid decrease in the FRET ratio from 0.46 at 1 min to 0.42 at 30 min (large PCL NCs). Consistently, most of the large PCL NCs quickly dissociated in zebrafish at 1 h post injection, while the large PEG-PCL NCs and small PEG-PCL NCs retained much higher integrity [58]. FRET imaging combined with zebrafish has also been used to study the in vivo behaviours of polymeric micelles using a co-loaded FRET pair, DiO and DiI [35].

FRET has been frequently used to monitor in vivo micelle integrity. Some FRET fluorophores suffer the self-quenching problem. To address this issue, Zhang et al. explored the large Stokes shift (LSS) fluorophores NBD-X and MS735 as the donor and acceptor, respectively. NBD-X and MS735 were separately conjugated to the hydrophobic block of the polyethylene glycol-block-poly(ε-caprolactone) (PEG-PCL) micelles to probe their stability. It is attractive to use fluorophores with a large Stokes shift of more than 80 nm for FRET study, or at least 55 to 80 nm to avoid self-quenching and light scattering in biological imaging, and thus with improved sensitivity and accuracy. The FRET experiments showed that despite the gradual dissociation of PEG-PCL micelles in blood circulation, about 60% of them remained intact in plasma at 72 h post i.v. injection [43].

In addition to polymer micelles, lipid NPs have also been investigated using FRET to monitor their integrity. Near-infrared probes, DiD and FP730-C18, were co-encapsulated in lipid NPs for in vivo imaging. At 1 h post injection, the FRET signal was observed throughout the whole body, suggesting the stability of the FRET-loaded lipid NPs. At 3 h, the FRET signal persisted but decreased in blood, indicating the presence of some intact lipid NPs. Additionally, the ex vivo organ imaging confirmed a strong FRET signal in the liver but a weak emission in the spleen. The biodistribution of lipid NPs at 5 h was found to be similar to that at 3 h. At 24 h, FRET was mainly detected from the intestine, demonstrating a biliary excretion pathway. In contrast, PEGylated lipid NPs exhibited prolonged blood circulation lifetime and improved stability in vivo with a subsequent accumulation in the intestine, skin, and ovaries [36].

**Figure 8:**
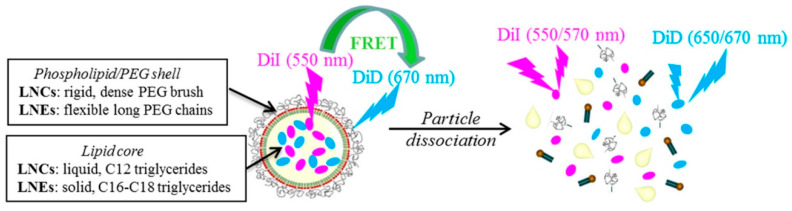
Schematic representation of the particles and the FRET process. Reproduced with permission from ref. [10]. Copyright 2014, American Chemical Society.

Lipid nanocapsules (LNCs) and lipid nanoemulsions (LNEs) were synthesized and studied for their stability. Gravier et al. co-loaded a FRET pair (DiI and DiD) in the LNCs and LNEs (Figure 8). LNCs with a capsule structure consist of a liquid core of triglycerides and a rigid shell. In contrast, LNEs have a solid core with low water solubility but a more mobile shell. Serum was very effective in destroying the two NPs. In comparison, LNCs could be internalized much quicker in cultured cells than LNEs, but LNEs dissociated rapidly after cell uptake. For in vivo studies, LNCs were found intact in tumour at 24 h post injection [10,36].

**Figure 9:**
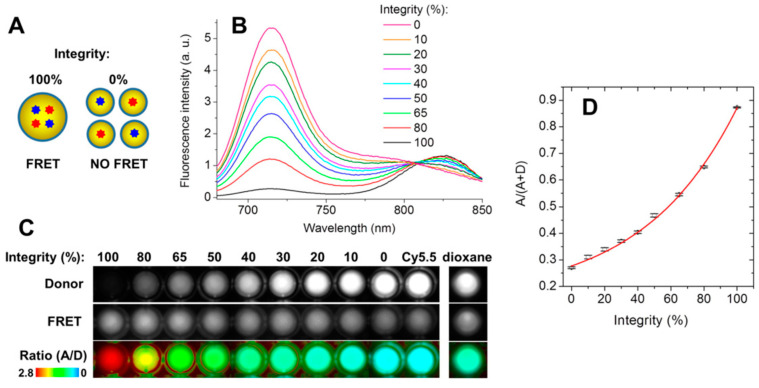
Calibration of ratiometric imaging using mixtures of intact FRET NCs with NCs containing donor and acceptor, respectively, at low concentrations (**A**). (**B**) Fluorescence spectra of NCs with different degree of integrity. (**C**) Fluorescence images of these mixtures. (**D**) Correlation between the A/(A + D) ratio and the level of integrity of NCs based on data in (**C**). Reproduced with permission from ref. [29]. Copyright 2016, the authors.

Many FRET studies are mainly qualitative in the early days. Ratiometric NIR FRET imaging offers a new strategy to realize the quantitative analysis of NP integrity in vivo. In contrast to absolute fluorescence intensity, the measured ratio values are independent of dye concentration and parameters of light sources, thus making them more reliable [29]. A lipophilic FRET pair, Cy5.5 and Cy7.5 dyes bearing long alkyl chains, was synthesized and encapsulated inside lipid nanocarriers. Then, lipid nanoemulsions (lipid NCs) were generated using dye-loaded oil and non-ionic surfactant (Cremophor^®^ ELP). As a semi-quantitative parameter, the FRET ratio, A/(A + D), only decreased slightly over 24 h incubation in serum, indicative of the relatively good stability of the FRET NCs [29]. To realize quantitative analysis of NP integrity, a calibration curve between the fluorescence efficiency A/(A + D) and NP integrity was derived (Figure 9). The NC signal at the tumour region was very strong after 2 h post injection with an NP integrity of 77%, which dropped to 40% at 6 h. The half-life of the NC integrity in tumour was 4.4 h, which was shorter than that in the liver of healthy mice (8.2 h). The lipid NCs maintained their integrity better, indicating that the NCs entered the tumour with minimal loss of integrity. It should also be noted that the NC disintegrated much faster in blood circulation of tumor-bearing mice than in healthy mice. This study provides solid evidence of the disintegration kinetics of lipid NCs in vivo, and shows that the lipid NCs preserved their integrity in the blood circulation, and entered tumours with good integrity, but they disintegrated hours after their accumulation in the tumors [29].

Based on the semi-quantitative correlation between the FRET ratio and the integrity percentage, Cayre et al. fabricated FRET NPs to monitor their stability over time. The FRET NPs were fabricated using the squalene–gemcitabine prodrug, and two derivatives of squalene with the Cy5.5 and Cy7.5, as an efficient NIR FRET pair. They remained intact in water after 6 h, and retained an 84% integrity after 24 h. Strikingly, the integrity of FRET NPs decreased to 30% in blood after 2 h incubation. After i.v. administration, they accumulated quickly in the liver, reaching the maximum accumulation with around 56% integrity at 35 min, and then a rapid disintegration with less than 10% of NP integrity at 2 h post administration [30].

Liposomes (LSs) and lipid disks encapsulating FRET dyes (DiO, DiI, and DiD) with different targeting ligands (CDX peptide, RGD peptide) were prepared to investigate whether they could traverse the blood–brain barrier (BBB) as intact forms. A BBB transwell model was established using brain capillary endothelial cells (BCECs). The lipid NPs remained intact when taken up by BCECs, as indicated by the FRET signal. After 3 h, 0.68‰ intact CDX–LS and 1.67‰ intact CDX–Disks traversed the in vitro BBB monolayer. Furthermore, the blood–brain tumour barrier (BBTB) was built using HUVECs on a transwell membrane, and 2.31‰ intact RGD–LS and 8.32‰ intact RGD–Disks passed the in vitro BBTB monolayer model. Similarly, liposomes and disks were observed to be capable of crossing the BBB and BBTB in vivo as intact forms.

**Figure 10:**
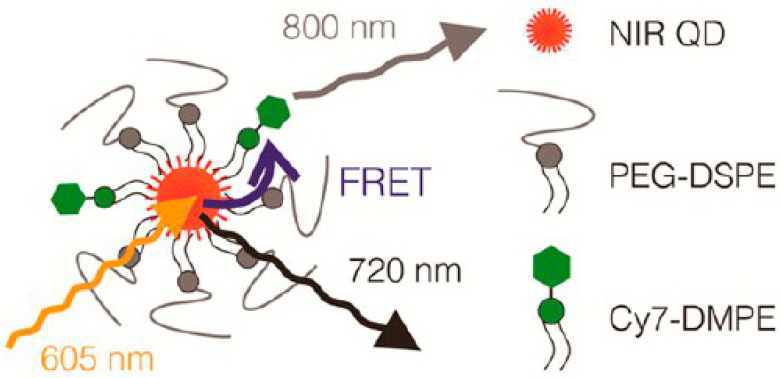
Schematic of lipid NPs that consists of a NIR QD core covered by a lipid coating (QD710-Cy7-PEG). Reproduced with permission from ref. [44]. Copyright 2013, American Chemical Society.

Quantum dot (QD) can also be used as a FRET fluorophore. Zhao et al. fabricated self-assembled lipid NPs using quantum dot (QD) and Cy7-labelled lipids to study their dissociation and tumour accumulation dynamics in vivo based on the FRET (Figure 10). Basically, the QDs were coated by a PEGylated Cy7-lipid monolayer via self-assembly. The QD serves as a FRET donor at an emission band of 710 nm and the Cy7-lipid functions as 800 nm-emitting FRET acceptor. The FRET between the QD core and the Cy7-lipid offers a sensitive method for the semiquantitative monitoring of the detachment of the lipid coating from the QD core. In the initial 2 h, the QD signal of the dual-labelled FRET QD710-Cy7-PEG increased faster and kept growing, indicating the dissociation of Cy7-lipids from the NPs, leading to the dequenching of QD emission [44].

NP stability and integrity are important properties for nanomedicine. However, it remained challenging to investigate NP integrity in vivo until the advent of the FRET technique. It makes the study feasible in both qualitative and quantitative ways. FRET ratio is a clear and informative parameter to measure and analyse. With the help of controlled NPs that carry donors and acceptors separately, quantitative analysis of NP integrity can be obtained.

## 6. FRET for Monitoring Drug Release

Fluorophores can be used as model drugs, so a FRET pair co-encapsulated in NPs could be used to monitor drug release. When a FRET signal is observed, this indicates the close proximity of both fluorophores in the core of intact NPs. Otherwise, the decrease in FRET signal would suggest the release of drugs and their dissociation from the NP core [10].

**Figure 11:**
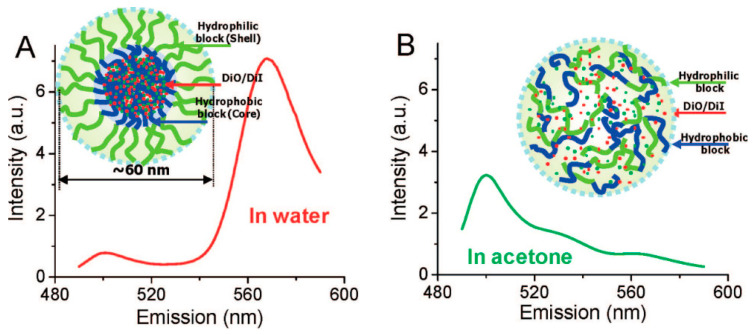
Fluorescence spectra of PEG-PDLLA FRET micelles diluted 10 times with water (**A**) and 10 times with acetone (**B**). Reproduced with permission from ref. [31]. Copyright 2008, American Chemical Society.

A FRET pair (DiO and DiI) was encapsulated inside the monomethoxy poly(ethylene glycol)-block-poly(D,L-lactide) (PEG-PDLLA) micelles to mimic drug release. An efficient FRET was observed in water with a very high FRET ratio of 0.9, and the FRET ratio decreased to 0.17 when the micelles were diluted in acetone (Figure 11). When the PEG-PDLLA micelles were injected in mice intravenously through tail veins, the FRET ratio rapidly dropped to 0.463 at 15 min post injection and then further decreased to 0.39 at 3 h, suggesting a quick release in circulation. Quick dilution of micelles could contribute to their disassembly, and thus drug release. However, the micelle concentration (38 µg/mL) in blood was much higher than their CMC (1.37 µg/mL). Further experiments proved that α- and β-globulins in serum were the key factors contributing to the fast dissociation of micelles in vivo [31].

**Figure 12:**
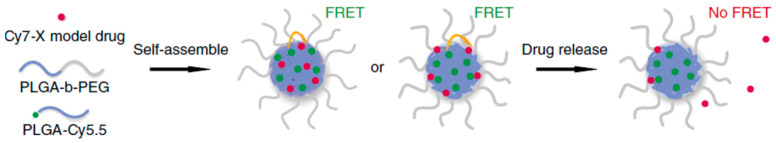
Schematic of the formation of Cy5.5-NP:Cy7-X FRET NP through self-assembly of PLGA(blue)–PEG(grey) block copolymer and Cy5.5(green)-conjugated PLGA in the presence of Cy7-X (red). Reproduced with permission from ref. [28]. Copyright 2016, the author(s).

Alternatively, polymers can be modified with a fluorophore, and the other fluorophore of the FRET pair can be encapsulated in the polymer NPs to investigate drug release. The block-copolymer PLGA–block-poly(ethylene glycol) (PLGA–PEG) NPs were formed through a co-self-assembly process with Cy5.5-conjugated high-molecular-weight PLGA as the core material, and Cy7 modified with different tails (carboxylic acid, C12, OLA, and PLGA2K) as model drugs (Figure 12). A linear correlation was observed between the average Cy7-X loading per NP and the measured FRET/Cy5.5 intensity ratio. Based on this linear correlation, it is possible to predict the amount of Cy7-X in the NPs. Increased FBS concentrations and higher temperatures resulted in quicker Cy7-Xs release. Their release rates are dependent on the hydrophobicity and miscibility of Cy7-X, following the order: CA > C12 > OLA > PLGA2k. The hydrophobicity and miscibility of drugs with the NPs are two independent key parameters that regulate drug loading and drug release, as well as their accumulation in tumours [28].

FRET is very useful for monitoring drug release, but it should be noted that FRET signals could also be from the transfer of lipophilic dyes from NPs to cell membranes, leading to a certain degree of FRET recovery. For example, lipophilic dyes released from polymeric NPs could rapidly transfer to cell membranes or intracellular organelles, resulting in a recovery of FRET, which compensates for the decreased FRET as a result of the dye release from NPs [32,37]. Consequently, the quantitative measurement of cargo release using FRET becomes difficult. To overcome this limitation, the FRET pair can be individually loaded into PEO-PCL NPs for FRET imaging. After DiD NPs and DiR NPs were co-administered in mice, no initial FRET signals were observed. Then, the later appearance of a FRET signal was mainly from the released DiD/DiR accumulated on the cell membrane surface and intracellular organelle membrane. Therefore, the increased FRET ratio in mice with co-administered DiD NPs and DiR NPs is more sensitive to cargo release. Additionally, compared to PEO-PCL NPs, poly(ethylene oxide)-b-polystyrene (PEO-PS) NPs exhibited slower cargo release, as indicated by the lower FRET signals when cultured with cells due to the high glass transition temperature of PS (107 °C), so the PS cores were very solid with low fluidity at 37 °C. It is generally assumed that polymeric NPs quickly disassemble in blood circulation due to dilution or interactions with plasma proteins and lipids, resulting in premature drug release. However, the NP concentration in circulation (0.2 mg/mL) was found to be much higher than the critical micelle concentrations (CMC) of PEO-PS (<1 μg/mL). Therefore, premature release in vivo was not due to the NP disassembly, but probably the in vivo sink effect of serum proteins, especially lipoproteins, as well as cell membranes [37].

**Figure 13:**
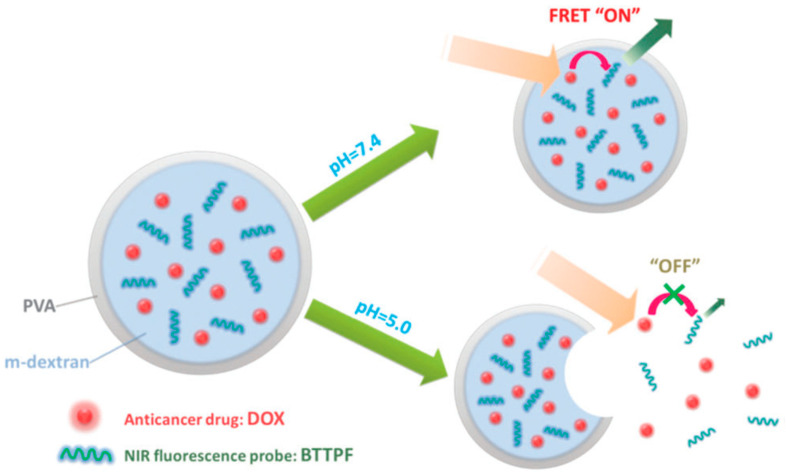
pH-responsive and NIR-emissive polymer NPs as carriers for anticancer drug DOX: fluorescence signal difference under mildly acidic or physiological conditions. Reproduced with permission from ref. [45]. Copyright 2014, The Royal Society of Chemistry.

To track doxorubicin release in vivo, a FRET pair was established using an NIR probe (acceptor BTTPF, emission 690 nm) and doxorubicin (donor DOX, emission 590 nm). BTTPE and DOX were coloaded in NPs made of pH-responsive dextran. At physiological pH (7.4), the FRET-pair-loaded NP showed a strong FRET signal, but it decreased by almost five times under acidic conditions (pH 5.0), indicating NP degradation (Figure 13). In vivo studies also demonstrated that BTTPF was able to monitor drug biodistribution and drug release in a non-invasive and real-time way [45].

**Figure 14:**
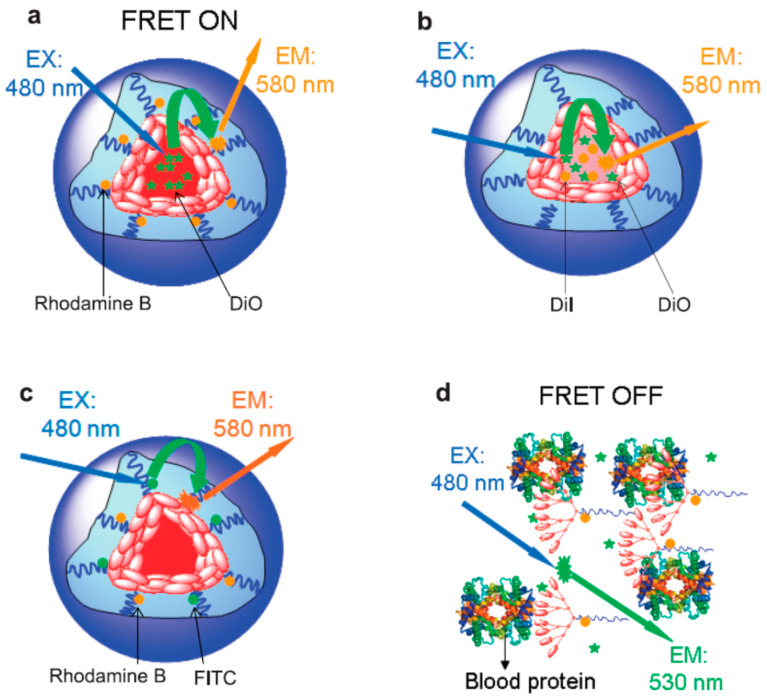
Schematic of non-cross-linked FRET-based NPs FRET-NCMN1 (DiO and rhodamine B pair) (**a**), FRET-NCMN2 (DiO and DiI pair) (**b**), FRET-NCMN3 (FITC and rhodamine B pair) in PBS (**c**), and FRET-NCMN1(DiO and rhodamine B pair) in human plasma (**d**). Reproduced with permission from ref. [32]. Copyright 2012, American Chemical Society.

Core-shell polymer NPs can be labelled using position-specific FRET pairs (Figure 14). Two polymer micellar NPs were synthesized via assembling polyethylene glycol (PEG)-block dendritic oligomer of cholic acid (CA) copolymers (called telodendrimers) (NCMN) and a cysteine-containing telodendrimer (DCMN). For the first position-specific FRET imaging, DiO (donor) was encapsulated in the core of NPs as a model hydrophobic drug and rhodamine B (acceptor) conjugated to the polymer to monitor the NP (FRET-NCMN1). The second FRET pair (DiO and DiI) was encapsulated in the core of NPs as a hydrophobic drug model to probe the drug release (FRET-NCMN2). The third FRET pair (FITC and rhodamine B) was conjugated to the polymer of the NP (FRET-NCMN3). The first FRET pair (DiO and rhodamine B) was also incorporated into the disulphide cross-linked NPs for comparison (FRET-DCMN1). The FRET signal of the three FRET reporter systems reflects the proximity between the model drug and the polymer, two different drugs, and different polymers within an NP, respectively. Both FRET-NCMN1 and FRET-NCMN3 NPs exhibited a dramatic decrease in FRET signal in human plasma within the first 30 min, with the FRET ratios decreasing to 33.8% and 29.5% at 1.5 h, respectively, indicating that human plasma can disrupt the non-cross-linked NPs [32]. However, the FRET signal of FRETNCMN2 changes slowly over 24 h in the presence of human plasma, due to the recovery of FRET as a result of the dye transfer from NPs to cell membranes and serum proteins. Further studies demonstrated that four types of lipoproteins (CM, HDL, LDL, and VLDL) contributed the most to the rapid decrease in FRET ratio of FRET-NCMN1, while serum albumin (HSA) and immunoglobulin gamma (IgG), showed a minimal effect on the FRET ratio. The disulphide crosslinked NPs can better retain their integrity with a slow drug release in serum [32].

Similarly, the FRET pair (DiO and DiI) was also co-loaded in deblock copolymer poly(ethylene glycol) (PEG) and poly(- propylene sulphide) (PPS) NPs for reactive oxygen species (ROS)-responsive drug delivery to alleviate sepsis-induced liver injury. The FRET signal was compared in healthy and septic mice at 2, 4, and 12 h post injection. Ex vivo imaging showed a significant decrease in the FRET signal in the sepsis mice compared to the healthy mice, indicating that the excess ROS accelerated the cargo release of the PEG-PPS-NPs, especially in the liver [59]. Chen et al. also encapsulated the FRET pair DiO and DiI in Schisantherin A (SA)-loaded mPEG−PLGA NPs (SA-NPs) for brain delivery targeted at Parkinson’s disease (PD). Zebrafish were used to investigate the in vivo fate of the NPs. The FRET ratio decreased from 0.84 at 1 h to 0.59 at 8 h post injection, illustrating the gradual dissociation of NPs in vivo. A low FRET ratio of 0.32 was still observed at 48 h, suggesting the existence of a small amount of intact NPs [33].

**Figure 15:**
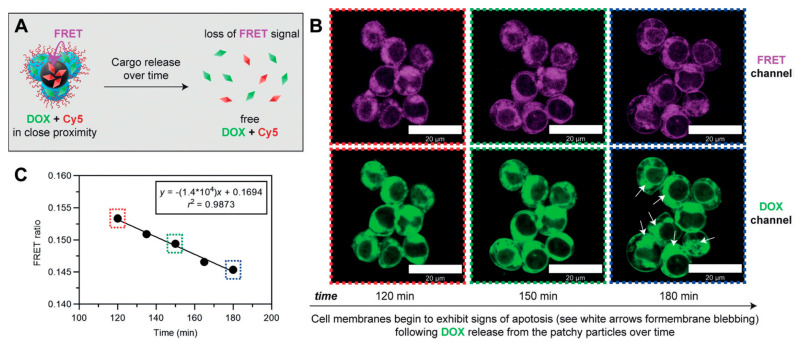
(**A**) Illustration of FRET mechanism of DOX/Cy5 co-loaded patchy particle. (**B**) Confocal images of MCF-7 cells at 120, 150, and 180 min incubation with DOX/Cy5 co-loaded patchy particles. (**C**) Changes in FRET ratio over time. Reproduced with permission from ref. [46]. Copyright 2019, Wiley-VCH Verlag GmbH & Co. KGaA, Weinheim, Germany.

FRET has also been explored to study drug release from patchy NPs using a linear ABC triblock terpolymer poly(glycosyloxyethyl methacrylate)-block-poly(-benzyl acrylate)-block-poly(4-vinylpyridine). DOX and Cy5 were loaded into the two compartments separately of the patchy NPs to probe real-time drug release. FRET signal from DOX to Cy5 was observed, likely occurring at the interfaces between the two compartments, i.e., core and patches (Figure 15) [46]. The FRET ratio declined linearly over time, suggesting the gradual release of the co-loaded DOX and Cy5 within the different compartments of the patchy NPs.

A new FRET pair (BODIPY-FL12 and Nile Red) was used to assess the stability of poly(lactic-co-glycolic acid) (PLGA) NPs. BODIPY dyes are good candidates for FRET due to their excellent stability. Additionally, the Förster distance between BODIPY-FL12 and Nile Red was increased significantly to 5.5 nm, enabling an efficient FRET as far as 11 nm (two times the Förster distance). FRET PLGA NPs remained stable for >2 weeks in buffers, but started to degrade in cells at 24 h with a complete FRET loss at 72 h. For in vivo mice experiments, PLGA NPs accumulated predominantly in liver with no FRET signal observed at 24 h post injection [47].

A J-aggregate-based FRET method was developed to elucidate the drug release of high-drug-loading NPs (50 wt%). A large red-shift (110 nm) occurs when a large number of single dye molecules are loaded in NPs, resulting in dye aggregation. Similarly, FRET signal red shifts (116 nm) when a FRET pair is loaded with high dye concentrations in the NP. Based on this J-aggregate-based FRET method, FRET-pair (DiO and DiI)-loaded polymer NPs demonstrated a two-step cargo release process intracellularly: firstly, the dissociation of the dye aggregates into dye molecules, followed by the dye release from the polymer NPs [19].

**Figure 16:**
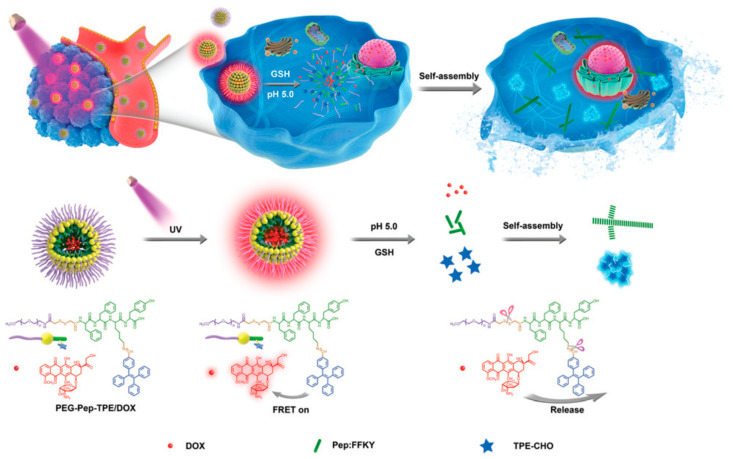
Schematic illustration of drug release behaviour from the self-assembled PEG-Pep-TPE/DOX NPs via FRET and pH/GSH-responsive antitumor synergistic chemotherapy. Reproduced with permission from ref. [48]. Copyright 2020, The Royal Society of Chemistry.

In addition to the use of dye molecules as drug surrogates, a real chemotherapeutic drug, DOX, has also been frequently used for FRET imaging due to its intrinsic fluorescent property. The FRET pair based on aggregation-induced emission luminogens (AIEgen, TPE-CHO) and the antitumor drug DOX was developed to detect the drug release of DOX-loaded polyethylene glycol-block-peptide (FFKY)-blocktetraphenylethylene (PEG-Pep-TPE/DOX) NPs. Upon the release of DOX from the NPs, the fluorescence of AIE fluorogen TPE-CHO is turned on (Figure 16) [48]. Li et al. reported a FRET polymer NP based on an anionic conjugated polymer PPEIDA with DOX through the metal coordination of Cu^2+^ with PPEIDA and DOX. PPEIDA−Cu−DOX CPNs exhibited high drug loading (54.3%) and encapsulation efficiency (95.80%). The FRET energy transfer from the polymer PPEIDA to DOX allows the real-time monitoring of drug loading and drug release [60]. Dong et al. developed a pH-responsive NP using DOX (the acceptor)-conjugated polymer PPEGMA20-PNAP8 (the donor) via a pH-responsive imine bond. The self-assembled PPEGMA20-PNAP8- DOX theranostic NPs displayed the FRET signal of DOX (orange fluorescence) indicating minimum drug leakage in normal physiological pH. In contrast, the fluorescence of the donor polymer PPEGMA20-PNAP8 recovered with a blue fluorescence after the pH-triggered release of DOX in the acidic tumour microenvironment [61].

Photothermal therapy (PTT) has been combined with chemotherapy using NIR-absorbing polymer (acceptor) coloaded with DOX (donor) to achieve a combinatorial anticancer capacity. The quenching of DOX in the NP as a result of the FRET between DOX and NIR polymer was used to probe DOX release intracellularly [62]. Other FRET pairs have also been developed to probe responsible drug release and degradation, such as FRET pairs using carbon quantum dots (CQDs) and DOX, nano-MnO_2_ and CQDs [49], aggregation-induced emission (AIE) dyes and DOX [22].

FRET has been widely used for monitoring the degradation and drug release of organic NPs (e.g., polymer micelles, polymer NPs, and lipid NPs). Yan et al. applied FRET for exploring the drug release of a smart-gated mesoporous silica NP (MSNs). A hypoxia-responsive azobenzene polymer was employed as the gatekeeper on the MSN NP surface. Coumarin 6 and rhodamine B were coloaded in the MSN NPs to explore the hypoxia-responsive cargo delivery [50].

Using FRET fluorophores as drug surrogates, or even directly applying fluorescent drugs such as DOX to investigate drug release, can provide non-invasive and real-time analysis. It significantly improves the knowledge of the release behaviours of drugs, especially intracellularly. Although the dye representatives may have many common properties with their mimicking drugs, their differences should be taken into consideration. How to determine the similarity between dyes and drugs and how to better simulate drugs using fluorescent dyes could be interesting to be explored.

## 7. Conclusions

Many FRET probes have been developed in monitoring NP drug delivery. Among all the probes, the DiO and DiI FRET pair is the most widely used ratiometric probe for investigating NP drug delivery, but mainly for in vitro studies. For in vivo studies, NIR dyes such as DiD and DiR are more suitable due to their short excitation wavelengths, minimal interference from background fluorescence, and weak tissue penetration. Compared with small-animal in vivo experiments (mice or rats), the zebrafish has gained increasing interest in exploring the nano–bio interactions of various NP systems. FRET ratiometric probes become more advantageous when using such transparent in vivo models. However, zebrafish models should be carefully designed to mimic the structure and function of certain biological barriers of mammals, and they should be properly validated to yield meaningful data.

To monitor the nano–bio interactions, many different strategies have been developed. A FRET pair can be positioned differently in NPs to explore their different properties. The donor and acceptor dyes can be co-loaded into the NPs as drug surrogates to monitor drug release. A strong FRET signal indicates a close proximity of these two dyes inside NPs, and the decrease in FRET suggests their gradual separation, and thus drug release. Alternatively, one dye can be encapsulated in NPs and the other dye can be conjugated to either polymers or lipids to label the NPs. In this case, the FRET signal represents the distance between the drug and NPs. In addition, the two dyes can be both conjugated to polymers or lipids, respectively, so the FRET signal provides more information about the dissociation or degradation of NPs. Furthermore, in addition to traditional fluorescent dyes such as lipophilic tracers and cyanine (Cy) dyes, new probes have been developed for FRET studies such as QDs, upconversion NPs, aggregation-induced emission dyes, and NIR polymers, offering new opportunities and advantages.

## Figures and Tables

**Table 1:** Common FRET pairs.

Donor	Acceptor	Donor Excitation (nm)	Donor Emission (nm)	Acceptor Excitation (nm)	Acceptor Excitation (nm)	References
Cy3	Cy5	555	569	651	670	[24]
Cy3.5	Cy5	579	591	651	670	[25]
Cy5	Cy5.5	651	670	683	703	[26]
Cy5.5	Cy7	683	703	756	779	[27,28]
Cy5.5	Cy7.5	683	703	788	808	[29,30]
DiO	DiI	484	501	549	565	[11,12,19,25,31,32,33,34,35]
DiI	DiD	549	565	644	665	[10,34,36]
DiD	DiR	644	665	750	780	[37,38]
FITC	Rhodamine B	495	519	546	568	[39]
Rhodamine B	Cy5	546	568	651	670	[40]
Curcumin	Hypericin	467	530	544	592	[41]
Fluorescein	Rhodamine B	498	517	546	568	[38]
Coumarin 6	DiI	457	501	549	565	[42]
NBD	MegaStokes dye 735	467	539	545	735	[43]
DiD	FP730-C18	644	665	741	764	[36]
QD710	Cy7	605	710	756	779	[44]
Doxorubicin (DOX)	BTTPF	470	560	595	690	[45]
DOX	Cy5	470	560	651	670	[46]
BODIPY-FL12	Nile Red	505	511	549	628	[47]
PPEGMA20-PNAP8	DOX	380	446	470	560	[48]
CQDs	DOX	365	435	470	560	[49]
H-PAMAM-ss-mPEG/α-CD	DOX	350	455	470	560	[22]
Coumarin 6	Rhodamine B	457	501	546	568	[50]

## Data Availability

Data sharing not applicable.
